# Rethinking and Reinforcing Cultural Humility Against the Culture Wars: A Framework For Addressing Receptivity to Diversity Initiatives

**DOI:** 10.1080/10872981.2024.2307710

**Published:** 2024-02-01

**Authors:** Jerel M. Ezell

**Affiliations:** Community Health Science, School of Public Health, University of California Berkeley, Berkeley, CA, USA; Berkeley Center for Cultural Humility, University of California Berkeley, Berkeley, CA, USA

Modern medicine is built upon white supremacist ideals and attendant policies that have precluded certain racial minority populations from accessing careers as physicians and other clinicians and also gravely diminished the quality of racial minorities’ health and healthcare [1,2] and yet, as the culture wars accelerate, such an empirically sound and salient statement has become a considerable source of polarization and animus inside and outside of the medical profession. Along these lines, the medical field can be said to suffer from the multilayered, iterative, and reproducing challenge of having both a dearth of physicians (and other clinicians) from underrepresented races as well as outsized, entrenched issues in validating and addressing the dynamic forces that foster race-related health disparities [3,4]. Indeed, Black and Latino individuals in the U.S., populations historically underrepresented as physicians, currently make up just 6.2% and 5.3%, respectively, of U.S. medical school graduates [5], contrasting sharply with the racial/ethnic diversity in adjacent fields such as social work [6]. Simultaneously, health outcomes for Black, I Latino, and Indigenous populations—particularly in regard to cardiovascular disease, most cancers, HIV/AIDs, asthma, and maternal-fetal health—are persistently much poorer than those of their white counterparts [7,8].

The broad, though often subtle, ways that physicians, directly and indirectly, have influenced and marshaled these inequities are well-known [2,9]. Contributions include the induction and substantiation of racial categories in the 18^th^ Century through medical pseudoscience and eugenicist theory down to explicit and implicit bias-driven clinical recommendations and unethical experimentation on minority populations to generate novel clinical techniques, therapies and medications [3]. However, a key omission in the literature in better contextualizing this backdrop has been a targeted focus on physicians’ receptivity to Diversity, Equity, Inclusion, and Belonging (DEIB) initiatives. In particular, there is a dearth of context on the acceptability of DEIB initiatives that aim to enhance physicians’ cultural humility, or their tendencies towards active listening, inquisitiveness, affirmation of cultural differences, and socioemotional capaciousness more broadly [10 and thereby diminishing these harms. In short, little has been theorized on the extent to which physicians are genuinely and authentically motivated, intrinsically or otherwise, to embace, practice, and manifest the principal ambitions of DEIB.

In 2021, the American Medical Association (AMA) issued updated guidelines meant to improve diversity in the U.S. physician workforce and position, albeit mostly symbolically, the medical field to better contextualize and address racial health disparities [11]. The report specifies approaches including “targeted recruitment and revised medical school admissions policies, curriculum changes, summer enrichment programs, and comprehensive programs that integrate multiple interventions, such as financial, academic, and social support.” [11] Guidelines such as these, while conceptually valuable, largely do not reckon with which ‘policies,’ ‘programs,’ ‘interventions,’ etc., related to the adoption of DEIB initiatives are actually most salient in terms of physicians’ likelihood of authentic engagement and good faith efforts toward incorporation into everyday practice. Indeed, the vast literature on medical trainings around DEIB demonstrates that physicians’ desire and compulsion to engage in DEIB efforts are typically presumed *a priori* to be fully or at least mostly manifest and very rarely empirically assessed or indeed even communicated as a notable mplementation challenge [12].

The persistence of racial disparities in health, buffeted by growing concerns around the specter of DEIB, academic freedom, and “cancel culture,” cast existing presumptions about physicians’ readymade acceptance of DEIB programming into sharp relief. Against the backdrop of present-day culture wars, many of those who wield power and influence in the healthcare system, including not just physicians but healthcare administrators, pharmaceutical companies, and health insurers, could be said to have a tendency, if they have any tendency at all towards DEIB intervention, toward only addressing what we may refer to as the “low-hanging fruit” of intervention. These low-hanging fruit correspond to an emphasis on scrutinizing and medicalizing *only individual-level* risk factors (e.g., health literacy, nutrition, etc.) rather than implicating structural forces such as systemic racism, community disinvestment, etc. Such approaches do not directly call into question or otherwise threaten institutional stakeholders’ power and also help ensure the ongoing commodification and economic furtherance of clinical practice, medical research, and so forth. Moreover, even in instances where there is earnest interest among healthcare’s powerbrokers to enact DEIB-related practices, the appropriate mechanisms (e.g., trainings, curricula, professional development time, etc.) to do so may not exist as a result of the conflicting priorities and reductive interests of administrators, health insurers, etc.

Presenting strategies aligned with cultural humility, this article seeks to contextualize and problematize physicians’ receptivity to DEIB initiatives centered on racial health equity, theorizing on physicians’ increasingly nuanced social positionality and their extrinsic and intrinsic motivations for pursuing medicines. The aim of this piece is to characterize the nature and viability of extant DEIB interventions in medicine and wider goals around enhancing representation in medicine and improving health outcomes among racial/ethnic minorities, emphasizing future directions and associated scaling potential.

## Strategy #1. Expand and enhance medical education through decolonization and cultural humility

Much of the focus on reversing the harms generated by deep, historically embedded structures of racial disenfranchisement in medicine and public health more broadly has focused on the so-called “decolonization” of medical education [13–15]. This upstream approach endeavors to instill a reflexive, multicultural perspective, and appreciation, in physicians at the earliest stage of their training (i.e., in medical school) by instituting pedagogy that is cross-cultural and referential in both philosophy and application. The decolonization process is accomplished by 1) de-elevating white and Euro-centric pedagogy that endorses hierachies, eugenics, and give primacy to dubious notions of there being manifest biological differences associated with race (e.g., in terms of morphology, pain sensitivity, morality, aptitude/intelligence, etc.) and 2) incorporating conceptual models, treatment schemas, case studies, etc. that are illustrative and responsive to all racial/ethnic identities and that, in turn, eschew one-size-fits-all modalities [16–18]. Presently, there are no universal, culturally humble guidelines outlining how such an approach can be implemented across medical schools or other medical education environments.

To this end, while an orientation toward decolonization is useful in terms of the individual “unlearning” regressive racialized concepts and medical constructs, this approach does not fully register the need for introspection and reflexivity in practitioners. Hence, it is necessary to consider not just forces such as implicit bias, a key, but amorphous, focal point in medical training, but also the extent to which physicians see themselves (and their colleagues, predecessors, and/or ancestors) as active perpetrators or passive facilitators of racial bias and of the structural disadvantage that the healthcare system reproduces. Part of the unlearning process would call for adjustment to the recalcitrance that may arise from physicians’ perception that they are not, directly or indirectly, part of this disenfranchising legacy and therefore do not need to engage in medical education decolonization efforts.

With this in mind, medical schools and healthcare systems’ education paradigms should more evenly coalesce around the idea of cultural humility [19,20]. Coupled with decolonization pedagogy, such an approach would speak to the need to understand and recognize one’s positionality through self-awareness exercises (e.g., asking how one “comes off” to peers, patients, etc.), active listening, being strengths-based and trauma-informed, thoughtfully considering of patients’ cultural context, their prior experience with institutional structures (including healthcare), and their expectations for clinical decision-making and care.

Modern medicine’s presumptive reorientation towards cultural humility calls for greater attention to structural and social determinants of health well-being—rather than delimited risk factors associated with patient behavior. Hence, the cultural humility movement must prompt a high degree of unlearning of siloed clinical conceptions of risk and causality that have heretofore been widely regarded as axiomatic [21]. Of note, this nascent transition to pedagogy that problematizes structural racism as a fundamental problem in medicine has already provoked considerable rancor and allegations of clinical “wokefication” and a chilling of academic freedom [22]. In recent months, medical schools across the U.S. have scaled back DEIB programming in view of rising public and political animus [23]. Rather than bend to this fervor, medical schools and healthcare leaders must double down, while acutely and unrepentantly framing DEIB programming as an apolitical matter of patient care. This renewed and lengthened effort should primarily focus on broadening and deepening how human ,morphology, beliefs, and (health) behavior is understood across racial/ethnic and intersectional axes, depoliticizing multicultural awareness and affirmation.

Along these lines, there is a broad need to target physicians’ readiness to disrupt conventional medical paradigms. Medical schools and healthcare systems can begin this process by instituting policy that incentivizes collaborative curricula creation between multicultural parties of physician-educators, social theorists, and publishers. Moreover, medical schools and healthcare systems can begin to invest in more culturally tactile and immersive forms of DEIB-focused learning, aiding in a reconceptualization of medicine’s ethos. Specifically, there is a need to pivot toward deeper, more adaptive cultural tailoring of case studies, namely as advanced through artificial intelligence (AI) and machine learning processes [24]. Such an approach can allow for more interactive case studies that enable learners to explore a diversity of cultural features and paradigms in a grounded manner that shifts from manipulable abstractions to praxis with cultural verisimilitude [25]. Such a digitally-scalable approach includes helping learners to more clearly visualize a racially diverse array of, for example, human anatomy (e.g., via interactive three-dimensional models and simulations on mobile phones or tablets). Moreover, particularly when driven by AI, the platform could allow for an endless variety of rich, culturally responsive exercises—e.g., allowing learners to listen to different styles of patient speech, accents, and body language and to experience scenario-based walkthroughs of different situations involving cultural tensions, etc. This orientation would provide learners with a more culturally attuned, visceral, practical, and immediate learning experience than traditional book-based curricula and in-class simulation can offer [26].

## Strategy #2. Understand physicians’ racial and sociocultural touchpoints by reinforcing academic freedom standards and fostering spaces for restorative justice

Cultural humility fosters the “concession” that healthcare is fundamentally political and its cultural ecology, as a result, is highly variable and formatively dependent on the political attitudes and dispositions of institutional power brokers. Healthcare practitioners, health insurers, and large medical associations such as the AMA and the Association of American Medical Colleges possess considerable clout in medicine, playing a deep and central role in creating distinctive “clinical climates,” in terms of establishing expectations and norms in interpersonal engagement *and* in formulating formal and informal policy that dictates how patients should be perceived and treated (and why), while tending to physicians’ reservations. Hence, insofar as it is crucial to attempt to, as the maxim goes, meet patients where they are, it is crucial to create space to reckon with where healthcare professionals are when it comes to understanding physicians’ politics and racial and sociocultural attitudes.

In explaining the synergies between the lack of racial diversity among physicians and the persistence of racial inequities in health, one first observes that power is highly concentrated among the wealthiest individuals in a society [19,20]. Insofar as there is a desire to maintain this power (and wealth), preservation efforts are made, with powerholders often utilizing mechanisms that directly infringe upon the health and well-being of populations with less social and political capital, namely racial/ethnic minorities. Along these lines, physicians in positions of power, and their administrative allies, will naturally lack earnest enthusiasm for committing to and enacting meaningful change in approaches to “level the playing field,” both in terms of diversifying their workforce and committing to racial equity-driven solutions to healthcare engagement with diverse patients.

In a recent Pew poll, 75% of Black people in America, versus just 46% of white people, said “more attention to the history of racism in the U.S. is good for society.” [27] In another poll, 59% of Black people in America versus only 25% of white people said that Black people are “treated less fairly than whites when seeking medical treatment.” [27] As socialized individuals, physicians, like others, possess beliefs and values that may not align with empirical evidence on racial disenfranchisement or the goals of DEIB trainings. It is recommended that medical schools and healthcare systems periodically administer anonymous surveys to clinical trainees and staff physicians to gauge their attitudes on the nature and extent of racial differentiation in the patient population, in the physician workforce, and in society writ large. Ongoing Pollyannaish treatment of physicians as staunch, readymade DEIB champions, or even as apolitical or “race-neutral,” either inside or outside of their workplaces, is both naïve and dangerous. Further, in alignment with “desirability bias,” [12] physicians are unlikely to acknowledge distaste for DEIB-oriented programming and theory (such as Critical Race Theory) or so-called “identity politics” (thus prompting the need for the aforementioned surveys to be anonymous).

Beyond concerns about improving individuals’ health, physicians’ primary motivations for a career in medicine frequently include higher-than-average salaries, the desire for unique and complex professional challenges, and heightened social status [28]. Thus, culturally humble DEIB education and training must also speak to the “non-altruistic” factors that have motivated individuals to enter medicine, and articulate the potential of DEIB to facilitate both personal and professional development.

In alignment with the aims of academic freedom, there is a need to develop policy to support physical or virtual spaces for physicians to announce social and political beliefs and misgivings about DEIB. This might consist of the creation of one-on-one “Peace Circles” where a neutral or external mediator can respond to concerns and work towards the co-creation of goals that will propel the physician towards personally-tailored DEIB approaches that do not compromise their values [29,30]. Restorative justice approaches like these that focus on mutual affirmation, rather than mediation toward a singular, circular set of “truths,” are more likely to be effective in helping physiciansnavigate and affirm differences [31]. Similarly, anonymous comment submission portals, structured around the articulation of one’s DEIB-related concerns and both pros and cons to redress of the concerns, can be used to guide team discussions.

To this end, frequently, practitioners are reluctant to participate in trainings because of concerns about overlap with and disruption of their ”real” duties, etc. This attitude, coupled with concerns about the clinical value of DEIB, further winnow interest in participation. Leaders and administrators in healthcare settings must thoughtfully present the case that cultural concerns *are* medical concerns and allocate (paid) time for physicians to participate in, and indeed even spearhead, DEIB programming. Similarly, healthcare systems should offer Continuing Medical Education (CME) credits to physicians to stimulate interest and involvement.

## #3. Develop strengths-based messaging on DEIB policy, programming, and physician potential

Efforts to address racial inequities equity in health are ultimately unsuccessful due to failures in changing the attitudes and behaviors of those who provide or facilitate care and powerbrokers who are are most “upstream” in the proverbial care cascade (e.g., lawmakers). This piece ends by considering that the incipient failure, however, is the failure in thoughtfully messaging DEIB interventions to attract and enroll physicians who need to be engaged. Recent public debates on the nature of systemic racism punctuate that a variety of opinions exist on the merits of claims that cultural, political, and economic systems, such as those undergirding healthcare, and by extension physicians, are or have been fundamentally racist [32]. In recent generations, physicians, at best, have been taught to adopt a functionally “colorblind” orientation to patients, this being the baseline expectation for cultural competence, cultural humility’s predecessor [33]. Under the cultural competence ethos, which eschews calls for practitioner self-awareness and dubiously stresses one’s capacity to “know” a culture that is not theirs, physicians are more likely to regard trainings that focus on culture as perhaps symbolically (or optically) important but ancillary to foundational clinical care. This mentality thus renders DEIB programming as fundamentally not a matter of medical import or medical ethics. Therefore, appropriate messaging of the purpose and value of cultural humility, as the genesis for generative involvement in DEIB, is of utmost importance.

With this in mind, physicians may feel offended by the implication that they harbor racist views or have implicit or unconscious biases that are harmful to the well-being of their racial/ethnic minority colleagues, patients, students, etc., causing them to reject the contentious messaging that often accompanies DEIB intervention [16]. Feelings of shame and guilt for dereliction are likely to be aggravated when physicians are mandated to attend such trainings, or are “voluntold” by supervisors to come, and subsequently feel that their (personal/academic) freedom is simultaneously being infringed upon or breached. These impediments to a positive or even neutral reception to calls for DEIB engagement highlight a need to be “strengths-based” in messaging. Strengths-based pedagogy emphasizes a need to consider the attributes, talents, and strengths that individuals naturally have or accumulate through lived experience, in lieu of, for example, an inordinate or myopic focus on the interrelational and communicaton-related limitations, weaknesses, and deficits of physicians [34,35].

This strengths-based paradigm can be superimposed onto our conceptualization of how physicians become motivated to enter the field of medicine; e.g., through exposure to impactful anecdotes that highlight the ways that physicians can both leverage existing skills and generate new ones toward the improvement of human health. In short, there must be a focus on addressing structural determinants of physician engagement—that is, the way DEIB is described, presented, and availed at the institutional leve—in view of individual-level dynamics that may otherwise diminish practitioners’ interest. If physicians are not sufficiently motivated by the way DEIB-focused interventions are articulated and offered by the institution, participation will ultimately be stunted.

Generally, speaking, participation in DEIB trainings centered on improving physicians’ engagement with racial/ethnic minorities may be curbed due to any one of the four following beliefs, namely that: 1) structural racism in healthcare does not exist, 2) structural racism in healthcare does exist but is not a meaningful problem vis-à-vis health outcomes, 3) structural racism in healthcare does exist but is not an addressable problem, and/or 4) that they (the professionally) are not (materially) racist and thus would not benefit from education or training on the subject. While the first three referents can be empirically debunked, the fourth requires amore nuanced response that can be addressed through focused messaging on the core importance of patient care.

Recruitment messaging that focuses solely on physicians’ “flaws,” as opposed to their potential and prospects for improvement, is less likely to engender thoughtful participation and sustained change in attitudes and behaviors. A strengths-based approach will continuously validate the importance of ongoing self-growth and learning as a professional and the improved productivity and success (in improving patient outcomes) that comes from this disposition [35]. Marketing of DEIB curricula and trainings that focuses on terms that may be regarded as buzzwords or culturally divisive—“decolonization,” “systemic racism,” “white privilege,” etc.—without granular explanation on context (e.g., that bias is common and wholly addressable), may elicit non-constructive emotions and ultimately serve as a deterrent to physicians’ earnest engagement. Likewise, it is critical to have a shift from platitudinous “virtue signaling” around DEIB contributing to an individual being a morally righteous and completely racially astute and harmonious physician to a focus on a physician constantly working on cultural humility.

In closing, cultivation of DEIB programming in clinical contexts must focus on both humanist and practical dimensions, with a particular emphasis on cultural humility as a competency and skill that enhances the overall socioemotional intelligence and capacity of the physician, reinforces their identity as providing high-quality care, and augments patient satisfaction and health through the increasing introduction of cultural congruence. A narrower, better calibrated concentration enables the physician to deliver more patient-centered care with attention to potential fissures in the physician’s cultural grounding and by extension that of their clinical practice. It is not enough to offer DEIB programming to physicians—interest and intentionality must be thoughtfully and purposefully stimulated and maintained in accord with physicians’ current and potential social embeddedness and their and others’ ever-shifting cultural tableaus.

## Abbreviations

AMA: American Medical Association
DEIB: Diversity, Equity, Inclusion, and Belonging
